# Grand challenges in insect systematics

**DOI:** 10.3389/finsc.2023.1327005

**Published:** 2023-11-28

**Authors:** Michael S. Engel

**Affiliations:** ^1^ Division of Invertebrate Zoology, American Museum of Natural History, New York, NY, United States; ^2^ Institut de Systématique, Évolution, Biodiversité (ISYEB), Muséum National d’Histoire Naturelle, Centre National de la Recherche Scientifique, Sorbonne Université, École Pratique des Hautes Études, Université des Antilles, Paris, France; ^3^ Museum at Prairiefire, Overland Park, KS, United States

**Keywords:** entomology, phylogeny, biodiversity, evolution, species discovery, homology, genomics, comparative biology

It is often said that systematics is the most synthetic of disciplines, the yoke that unites seemingly disparate fields of biological exploration. The truth of this assertion has only grown deeper roots through time. Once perceived as either largely equivalent to alpha taxonomy or alternatively, to phylogenetics, systematics is far richer. In fact, paraphrasing a common aphorism, nothing in biology makes sense except in the light of systematics. Whether it is understanding the origins and spread of pathogens, the geographic and ecological occurrence of species, the appearance and function of evolutionary novelties, the shifting of phenotypes to habitats, or the rise and loss of diversity, some core element of systematics is at play. The launch of the Insect Systematics section for *Frontiers in Insect Science* is therefore of considerable importance.

It is so oft repeated that insects are the most diverse lineage of life that the sentiment has become a cliché. Yet, it is important to understand that this axiom is less about a large number of species or individuals but is more an assertion of the grand challenge that is set before entomologists. It is one thing for 10,000 scholars to attempt a comprehensive understanding of 6,399 extant mammal species, while it is wholly another for the same number of researchers to grasp the over 1,150,000 species of insects, and terrifying to consider possibly a further four or more million living species and potentially hundreds of millions throughout their long evolutionary history. Such diversity is enough to overwhelm, leaving many to exasperation and exhaustion. Others attempt to simplify the problem by putting up blinders to the daunting extinct diversity or to groups beyond a narrow specialty, losing the explanatory power that comes from a breadth of vision. If we be bold, though, an unexplored land awaits and one that will continually enthrall and never run short of surprises. Entomologists should never be territorial or competitive for there shall always be more than any one or few of us can hope to explore. The section for Insect Systematics, in conjunction with its associated Loop platform, strives to become a place for open communication, collaboration, and publishing of the finest aspirations of the field.

Those who study the systematics of vertebrates, plants, or virtually any macroscopic lineage beyond the bounds of the Arthropoda find it difficult to understand the challenges of insect systematics, both exciting and daunting. The most easily perceived hurdle is the constant stream of new species to be discovered in the far-flung regions of the world, and sometimes even in the less exotic environments of our own backyards (1). While some fields consider the discovery and description of new species to be a thing of the past or a minor element of the field (*e.g*., mammalogy, ornithology), most insects are yet to be formally documented by science, and so, taxonomic science is vital. Proper taxonomic research includes support for fieldwork as such exploration is more critical than ever as human impacts leave our habitats fragmented, toxic, or outright destroyed along with their thousands of unique species (2). It is true that some of these “new” species may be known to indigenous peoples, alongside traditions of exceptional knowledge, but it is nonetheless necessary to complete a scientific account of such species alongside the multitude of “unseen” species—new to not only the scholar but also those living in the same region. Where possible, the formalized documentation of new species can bring together ethnological elements, should they exist. Most of our new species, however, fall outside of such bounds, and therefore, a greater investment is required for fieldwork and publishing taxonomic accounts and revisions, coupled with high-resolution images of specimens in the field and lab (2). There are tremendous opportunities available to taxonomic science today, particularly in visualizing morphological data—from 3D morphometrics and phylomorphometry to nanoscale CT scans (3–5). As these technologies become more readily accessible, they shall reshape our descriptive sciences, which are those upon which all other branches of evolutionary biology are built. These same tools are shining a new light on historical material preserved in museums, which are in essence the laboratories of biodiversity science. Biological research collections in museums throughout the world are dynamic laboratories for original discovery, particularly now when we can deploy techniques that were inconceivable a mere decade ago. The formal scientific discovery and documentation of new species are a huge undertaking for insect systematics and must be as robust today as any other subdiscipline of systematics. Seemingly “invisible” actors keep our forests blooming, producing oxygen, shelter, and food. Entomologists cannot shirk their responsibilities in this enterprise while increasing numbers of species slip into extinction each year. Insects are fundamental to the basic mechanisms of life on Earth; failure to learn even the most trivial of their biologies endangers us all. It is not hyperbole to claim that insect systematics has become a life-or-death effort as we watch the acceleration of our growing climatic upheaval, seem incapable of stemming the erosion and toxification of our environments, allow invasive species to spread unchecked, and stand stupefied as the world burns. This challenge, which starts with taxonomic discovery, must be met or we all lose.

For the “mere” million plus species already known, deep exploration of individual characters and character systems is becoming more prevalent in insect systematics. Comparative morphological studies, whether or not coupled with phylogenies, are necessary as the first step toward breaching the barrier that currently impedes much functional explanation of characters that are seemingly critical to the success of clades at all scales. An excellent example of such work includes the recent monograph of tracheation across apterygotes, paleopterans, and Polyneoptera, rich in CT data for all of the pertinent orders (5). As the empress of the sciences, such deep dives into morphological systems—particularly those other than traditional character suites—will revolutionize our understanding of insect evolution and ecology. With such work, we can similarly tackle an even more fundamental element: how we talk about insect evolution. Much of the language surrounding such characters—key innovation, functional trait, and the like—is ambiguous at best and some of the literature assumes that any character that can be mapped to the basal node of a particularly successful clade must therefore be a critical “novelty”, “adaptation”, or “key innovation”. Instead, this should be seen as a first step and that these are merely working hypotheses [and from published data most “adaptations” in insects would not constitute adaptations at all but instead exaptations: (6)]. As a field, we must go deeper in our understanding and create truly testable and comprehensive criteria before we slap on such labels. Entomologists have always been leaders in the development of new concepts and methods, and here too, they can lead the way. Insects are champions of diversity, and so, offer our best hope of clarifying evolutionary concepts, as examples of every variety abound. Moreover, while it is nice to identify an individual trait as key to the success of a given clade, rarely is biology so easily distilled to such soundbites—regardless of how nice they may make titles for papers. Do we lose out on a fundamental understanding of evolution by seeking oversimplified explanations, failing to do due diligence in finding the progression and interaction of numerous characters and historical events, biotic and otherwise, that lead to success? What even is *success*? For example, claiming that a clade diversified and succeeded because of coevolution with flowering plants is a classic example of oversimplification—it will catch the eye of readers, but it tells us only the tiniest fraction of what may have occurred and how we understand it. Success or failure in evolution is only achieved by the interplay of numerous characters (morphological, behavioral, physiological, genetic, etc.), a plethora of stochastic events, interactions among vast numbers of species, and the changing vicissitudes of climate drawn out over thousands if not millions of generations and hundreds or more speciations (and an even greater number of extinctions). If our goal is to understand evolution, then we must dive beneath the waves rather than continue to skip rocks across the surface. The new Insect Systematics section welcomes contributions that seek to improve concepts in evolutionary biology.

Naturally, beyond the comparative morphology and function of traits, as well as their phylogenetic distribution and character-state transitions, a fundamental knowledge of morphogenesis is required. Embryology has a long tradition in insect systematics, particularly for holometabolous insects where the larval and adult stages live such disparate lives. The exploration of immature insects is vital, whether in terms of documenting developmental stages or understanding the parameters (genetic and otherwise) in embryogenesis. Developmental and genome biology are vital subsets of systematics, exploring the link between genotype and phenotype, the influence of environment and behavior on specific phenotypes, and how morphogenesis has changed in the course of evolution (7, 8). Evo-devo is already a robust field, but too often it plays close to home—fearful of venturing phylogenetically distant from model organisms. This is understandable given the challenges and costs of establishing novel model systems. Nonetheless, playing close to shore will only get us so far, and in a group as massive as insects, the greatest rewards will be for those who are brave, venturing far from our few phylogenetic landmarks. Some will fail, but this should not be a barrier to exploration. Likewise, genome biology is massive, but for insects, it remains too fragmented. It is true that vast genomic data are available and that these will continue to expand, but the application of multiomics has progressed slowly in insect systematics. Additionally, while genomes are published and comparative genomic works are available, a truly comprehensive comparative genomic treatment across Hexapoda is lacking or at least, remains rudimentary. Much is to be gained by tackling a “monograph” of comparative genomics for insects.

Perhaps one of the more interesting venues for current and future exploration are those traits I have referred to in lectures as *heterologs*. Heterology is that system of partial homology accounted for with deep homologies whereby the underlying genetic architecture is homologous but the morphological phenotypes produced are independent as evidenced by both phylogenetic distance as well as morphological composition and developmental expression, both homologous and yet not at the same time. A term like deep homology (9, 10) emphasizes the shared genetic basis but what is perhaps more interesting is how in unrelated clades the phenotypes are independent. Heterology places its focus on the independent phenotypes, while recognizing the underlying, deep genetic homology. Although a classic example of heterology are the eyes of octopods and vertebrates (11, 12), there are tremendous instances among insects such as the gills, gin traps, and prothoracic horns of mayflies, tenebrionids, and scarabs (13–16). How many unrecognized heterologs remain to be discovered across a group as diverse as insects? The co-option of the same genetic architecture to produce unrelated and different phenotypes allows a more limited genome to express greater novelty. Yet, precisely how does such different expression originate in microevolutionary scenarios and produce such diversity over macroevolutionary time? Such changes are not to be conflated with *cyclical homologies*, or those homologous phenotypes that are repeatedly lost and regained across a clade (*e.g*., wings in Phasmatodea, male tarsal expansions in Megachilidae). We are only beginning this voyage.

Phylogenetics is too often regarded as equivalent to systematics, rather than the mere subset that it is, and is already a robust field. New phylogenetic estimations for insects appear by the dozens each month and based on everything from morphology to traditional DNA sequences to transcriptomes and ultra-conserved elements. These analyses are also progressively more encompassing, with increasingly larger numbers of species represented. Accordingly, the potential for these estimates to build hypotheses of historical patterns—biogeography, host associations, times of divergence, and origins and changes in biological phenomena, among a few—has only become more robust and pervasive. Yet, at the same time, these are poised to have their value tarnished as works become more boilerplate. Our phylogenetic studies are more numerous and complex in methodology, but the questions, and through them the answers achieved, are beginning to stagnate. Perhaps this trend is due to a tendency to mine for data rather than seek out or generate new sources of information. Our challenge is to more fully integrate our phylogenetic exploration with the aforementioned fields of inquiry, reinvigorating insect phylogenetics.

Naturally, the ultimate expression of systematics is the synthesis of each of the above examples, along with a plethora of others too numerous to include. The systematic study of insects offers more to the intrepid researcher than any other lineage, an indulgent salmagundi of possible intellectual treats. The meal is before us and all we need to do is take a bite. Come enjoy the feast with the Insect Systematics section of *Frontiers in Insect Science*.

## Author contributions

ME: Conceptualization, Writing – original draft, Writing – review and editing.
